# Analysis of an extended chromosome locus 2p14–21 for replication of the 2p16.3 association with glaucoma susceptibility

**Published:** 2011-04-29

**Authors:** Kyunglan Kim, Yong-jun Yun, Sewon Kim, Jong-Sung Kim, Chang-sik Kim, Changwon Kang

**Affiliations:** 1 Department of Biological Sciences, Korea Advanced Institute of Science and Technology, Daejeon, Korea; 2 Department of Ophthalmology, Chungnam National University Hospital, Daejeon, Korea; 3 Department of Family Medicine, Chungnam National University Hospital, Daejeon, Korea

## Abstract

**Purpose:**

Susceptibility to primary open-angle glaucoma (POAG) has recently associated with three intergenic single-nucleotide polymorphisms (SNPs) on human chromosome 2p16.3, just outside of the POAG-linkage locus GLC1H (2p15–16.2), in an Afro-Caribbean population. Especially, association of one SNP (rs12994401) was very strong (odds ratio 35) and later replicated in Afro-Americans but not in Ghanaians or Japanese. An extended region was examined in this study to look for SNPs of cross-population association.

**Methods:**

The three reported SNPs and all 63 SNPs considerably correlating with rs12994401 (r^2^≥0.3) in the African-descendent Yoruba were examined for POAG susceptibility association in a Korean population of 1,159 unrelated participants including 226 cases with glaucoma. As these 66 SNPs were spread from 2p14 to 2p21, all SNPs in this extended region were imputed for susceptibility association tests.

**Results:**

No susceptibility association was detected with rs12994401 in comparisons between 933 controls and 188 POAG (or 175 high-tension glaucoma) cases (statistical power of 100%), as well as with all 19 other typed SNPs, using logistic regression with adjustment for age and gender. The other 46 SNPs were deemed non-polymorphic in Koreans. Among 21,201 SNPs located in 2p14–21, only 4,260 were imputed to be non-monomorphic, but none of them passed a significance level of multiple testing. No association was observed when the samples were stratified by age or gender.

**Conclusions:**

No typed or imputed SNPs within 2p14–21 showed association with susceptibility to POAG, suggesting that the population inconsistency in 2p16.3 association was unlikely due to linkage disequilibrium differences.

## Introduction

Glaucoma is the leading cause of irreversible blindness [1], resulting from optic nerve degeneration. It is characterized by gradual death of retinal ganglion cell axons, which enlarges the optic-nerve cup, leading to visual loss. However, the two main types of glaucoma differ from each other in the appearance of the iridocorneal angle, where the base of the iris attaches to the peripheral cornea and sclera. The angle, which is the site of aqueous drainage from the anterior chamber, is open in the primary open-angle glaucoma (POAG), the most prevalent type. In contrast, in the primary closed-angle glaucoma (also known as primary angle-closure glaucoma), the angle is closed and the iris contacts trabecular meshwork, obstructing outflow of the aqueous humor from the eye, and consequently elevating intraocular pressure (IOP).

Elevated IOP is one of the strongest risk factors for apoptosis of retinal ganglion cells [2], being the only factor proven to be treatable. Whereas IOP is elevated in all primary closed-angle glaucoma, however, IOP is elevated only in some POAG (>21 mmHg, high-tension glaucoma) but remains normal in others (≤21 mmHg, normal-tension glaucoma) like unaffected individuals. Thus, glaucoma is fairly heterogeneous, and etiologies would differ from subtype to subtype, although the genetic and environmental factors contributing to the disease etiology and progression are not fully characterized yet.

Nevertheless, family history is another strong risk factor [3], suggesting that genetic factors contribute quite substantially to glaucoma etiology. Several linkage analyses revealed 21 POAG susceptibility loci, including 14 assigned as GLC1A through GLC1N [4] and 7 unassigned yet [5,6]. Several genes located within these loci have been identified to associate with POAG; for example, genes for myocilin (*MYOC*) in GLC1A [7], optineurin (*OPTN*) in GLC1E [8], and WD repeat-containing protein 36 (*WDR36*) in GLC1G [9]. The penetration of each gene to the disease is very low (1%–5%), however, and there are still more genetic factors to be disclosed.

**Figure 1:**
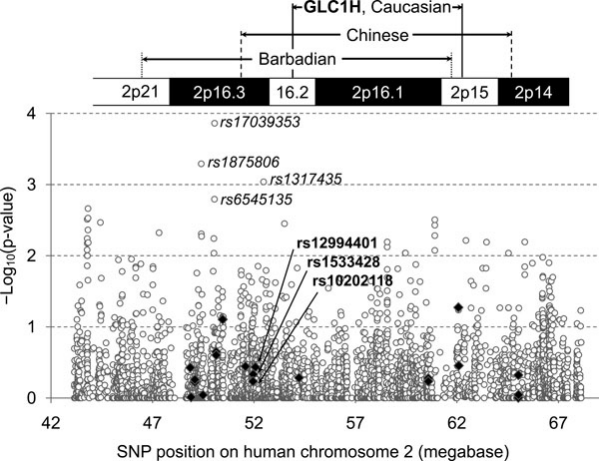
POAG susceptibility association tests for 4,280 SNPs on human chromosome 2p14–21. Negative logarithms (base 10) of p-values (y-axis) calculated for SNP association with susceptibility to POAG using the logistic regression analysis with adjustment for age and gender are plotted against the SNP chromosome positions in megabases (x-axis). These SNPs span 25 megabases from chromosome position 43,146,705 (chromosome 2p21) to 68,145,622 (2p14), and include the 20 typed (filled diamond) and 4,260 imputed ones (empty circle) that were not monomorphic. This region encloses the three POAG-linkage loci previously reported for Caucasian, Chinese, and Barbadian families. The three typed SNPs previously reported for the Barbadian association (in bold) and the four imputed SNPs showing the lowest p-values (italicized) are marked by their reference SNP accession ID's (rs numbers).

The eighth POAG-linkage locus, GLC1H, was mapped on human chromosome 2p15–16.2 as linked to adult-onset POAG in Caucasian families [10] (Figure 1). Later a broader region (2p14–16.3) was linked to juvenile-onset POAG in Chinese families [11] and yet another broader region (2p15–21) to adult-onset POAG in Afro-Caribbean families [12]. With no genes within or around GLC1H identified for glaucoma association yet, three intergenic single-nucleotide polymorphisms (SNPs) located on chromosome 2p16.3, just outside of GLC1H, have recently associated with susceptibility to POAG in an Afro-Caribbean population of Barbados, West Indies [12]. However, the association was replicated with only one (rs12994401) of the three SNPs in an Afro-American population [13], but with none of them in a Ghanaian [13] or a Japanese population [14].

The Barbadian association was replicated with none of the three SNPs in a Korean population in this study with statistical powers reaching 100%. Among many other possible causes for inconsistent or contrasting association results in different populations, we then explored a possibility that the inconsistency arose from differences in the structure of linkage disequilibrium (LD) among the tested populations, despite of the very high odds ratio (OR=35) of POAG association with rs12994401 in the Barbadian population. We hypothesized that a real causative variation(s) should considerably correlate with rs12994401 in Barbadians, but with other SNPs or haplotypes in Koreans—these SNPs correlate with rs12994401 considerably in Barbadians but not necessarily in Koreans.

All SNPs correlating considerably with rs12994401 in an African-descendent Yoruba population were spread in a broad chromosomal region from 2p14 to 2p21, which in fact enclosed all three reported POAG-linkage regions around GLC1H (Figure 1). Many SNPs in 2p14–21 were either genotyped or imputed for all participants of this study for testing association with POAG susceptibility.

## Methods

### Study subjects

All 1,159 participants were unrelated Koreans who were recruited from the Chungnam National University Hospital (Daejeon, Korea), and each and every participant provided written informed consent in this study that was approved by the Institutional Review Board of the hospital following the tenets of the Declaration of Helsinki. Each patient's diagnosis was confirmed and finalized by a glaucoma specialist (C.-S.K.), who reviewed all of the IOP measurements, morphology of the optic nerve head, retinal nerve fiber layer photography, gonioscopy, and automated visual field test results, at the glaucoma clinic of this tertiary referral hospital. Some subjects were clinically monitored for up to 12 months to exclude glaucoma suspects. Among 226 cases with glaucoma, 188 cases (83%) had POAG (including 175 high-tension and 13 normal-tension POAG), and only 38 cases (17%) had primary closed-angle glaucoma (including 30 acute and 8 chronic closed-angle glaucoma). All other types of glaucoma including congenital and secondary glaucoma were excluded from the case group. The 933 controls were free of any ocular and systemic diseases, and all had normal IOP levels (≤21 mmHg). Those who had glaucoma family history or steroid treatment were excluded from the control group.

### Genotyping

Genomic DNA were extracted from the whole blood samples of all subjects using the Gentra Puregene Blood Kit purchased from Qiagene Korea Ltd. (Seoul, Korea), and they were genotyped using the iPLEX Gold assay on the MassARRAY^®^ platform of Sequenom, Inc. (San Diego, CA) according to the manufacturer’s instructions, as previously described [15], with an approval from the Institutional Review Board of Korea Advanced Institute of Science and Technology. Amplification and extension primer oligonucleotides that were used in the multiplexing iPLEX Gold assay reactions were designed using the SpectroDESIGNER 3.1 program (Sequenom) and purchased from Bioneer Corp. (Daejeon, Korea). Individual genotypes were called using the SpectroTYPER 3.4 program (Sequenom).

### Imputation

A total of 21,201 SNPs located in a chromosomal region of 25 megabases from position 43,146,705 (on chromosome 2p21) to 68,145,622 (on 2p14) were imputed for all Korean participants by matching their genotype data of the 24 typed SNPs with the phased haplotype data for the Chinese (CHB) and Japanese (JPT) populations (2n=180 haplotypes) of the International HapMap Project using the MaCH v1.0.16 program [16]. The MaCH program was run with 100 iterations of the Markov chain model in two steps (mach1 -d sample.dat -p sample.ped -s chr2.snps -h chr2.hap --compact --greedy --autoFlip -r 100 in the first step; and mach1 -d sample.dat -p sample.ped -s chr2.snps -h chr2.hap --compact --greedy --autoFlip --errorMap par_infer.erate --crossoverMap par_infer.rec --mle in the second step).

### Susceptibility association tests

Genotypic association with susceptibility to POAG, high-tension POAG, or glaucoma was statistically tested for all 20 SNPs that were typed to be polymorphic, primarily according to a co-dominant genetic model using multivariate logistic regression analysis with adjustment for age and gender, unless specified otherwise. The significance level was α=0.05/20=0.0025 following the Bonferroni correction for multiple testing. All 4,260 SNPs that were imputed to be non-monomorphic were tested in the same way but with a significance level α=0.05/4260=1.2×10^−5^. For very rare SNPs where no minor-allele homozygotes were present, the heterozygotes were compared only with the major-allele homozygotes with adjustment for age and gender instead of following a co-dominant genetic model. Association was considered marginal when α<p≤0.05. Hardy–Weinberg equilibrium was assessed for each typed or imputed SNP for the control subjects. All statistical tests were performed using the PASW Statistics v.18.0 (SPSS Inc., Somers, NY) or PLINK v.1.07 package.

## Results

Three intergenic SNPs (rs12994401, rs1533428, and rs10202118) on human chromosome 2p16.3 have recently associated with susceptibility to POAG in an Afro-Caribbean population of Barbados, West Indies [12]. The reported odds ratios were high (35, 6.7, and 2.0, respectively) enough to be reliably tested in this study using 226 unrelated Korean subjects with glaucoma and 933 ethnicity-matched control subjects (whose characteristics are shown in Table 1), as the statistical power reached 100%. All participants were genotyped for the three SNPs with call rates ranging from 94% to 99%, and all were under Hardy–Weinberg equilibrium.

**Table 1:** Characteristics of the study participants.

**Characteristics**	**Glaucoma**	**Controls**	p*
**Demographic characteristics**	**n=226**	**n=933**	
Gender: male/female, no.	132/94	473/460	0.038
Age, year	60.9±13.6	57.6±13.2	<0.001
Body mass index, kg/m^2^	23.5±3.41	24.2±4.25	0.17
Systolic pressure, mmHg	129.4±16.7	129.5±24.0	0.94
Diastolic pressure, mmHg	79.2±9.4	77.8±11.4	0.054
**Clinical characteristics**			
Intraocular pressure, mmHg	26.4±10.4	15.3±2.8	<0.001
Cup-to-disc ratio	0.83±0.17	0.41±0.05	<0.001
Visual field mean deviation, dB	−10.7±10.1		
Visual field pattern SD, dB	6.13±4.19		

Data are expressed as the mean ± standard deviation (SD). *The glaucoma case and control groups were compared using Student's *t*-test.

The SNP association with glaucoma susceptibility was tested using multivariate logistic regression analysis with adjustment for age and gender, because these distributions were not matched between the case and control groups, whereas body mass index and systolic and diastolic pressures were matched (Table 1). The case recruitment in this study was intentionally biased toward POAG (83.2% of all glaucoma cases) to match with the Barbadian cases in the original study (all POAG), and toward high-tension glaucoma (77.4% of all cases) to match the average IOP level (26.4 mmHg) with the Barbadian cases (26.7 mmHg). Accordingly, the association tests were performed primarily with respect to susceptibility to POAG and high-tension glaucoma (Table 2).

**Table 2:** Association tests for three typed SNPs on 2p16.3.

Group*	**Genotype no.**	**Co-dominant model, p†**	**Dominant model**		**Statistical power‡**
** **	** **	** **	**OR (95% CI)†**	**p†**	** **
rs12994401** (*C*>*T*)**	***CC*/*CT*/*TT***	** **	** **	** **	** **
Controls (n=918)	376/422/120	–	1 (reference)	–	–
Glaucoma (n=222)	101/90/31	0.29	0.82 (0.61–1.10)	0.18	100%
Open-angle	81/76/29	0.37	0.89 (0.64–1.22)	0.46	100%
High-tension	76/70/27	0.36	0.87 (0.63–1.22)	0.42	100%
Normal-tension	5/6/2	0.97	1.10 (0.36–3.40)	0.87	–
Closed-angle	20/14/2	0.13	0.51 (0.26–1.01)	0.054	–
rs1533428** (*G*>*A*)**	***GG*/*GA*/*AA***	** **	** **	** **	** **
Controls (n=923)	355/443/125	–	1 (reference)	–	–
Glaucoma (n=222)	96/102/24	0.37	0.82 (0.61–1.11)	0.20	100%
Open-angle	78/87/19	0.46	0.85 (0.62–1.18)	0.33	100%
High-tension	74/81/16	0.31	0.82 (0.59–1.15)	0.25	100%
Normal-tension	4/6/3	0.59	1.41 (0.43–4.63)	0.58	–
Closed-angle	18/15/5	0.59	0.71 (0.36–1.37)	0.30	–
rs10202118** (*T*>*C*)**	***TT*/*TC*/*CC***	** **	** **	** **	** **
Controls (n=874)	333/413/128	–	1 (reference)	–	–
Glaucoma (n=212)	88/99/25	0.49	0.86 (0.63–1.18)	0.35	86%
Open-angle	71/85/20	0.58	0.90 (0.64–1.26)	0.53	68%
High-tension	68/79/17	0.39	0.86 (0.61–1.21)	0.38	60%
Normal-tension	3/6/3	0.50	1.84 (0.49–6.85)	0.36	–
Closed-angle	17/14/5	0.66	0.73 (0.37–1.44)	0.36	–

*All cases had primary glaucoma of open-angle (including high- and normal-tension subtypes) or closed-angle type. †Odds ratio (OR), 95% confidence interval (CI), and p-values were calculated in comparisons of glaucoma or subtype cases with controls using logistic regression with adjustment for age and gender. ‡Statistical power was calculated for the reported odds ratios [12] using the CaTS method [18], which is applicable only to groups of 100 samples or more.

No significant difference was observed in the genotype distributions of all three SNPs between the POAG case and control groups according to a co-dominant genetic model (0.37≤p≤0.58). Additionally, the differences were not significant between the control group and the high-tension POAG subgroup (0.31≤p≤0.39). The subgroups of normal-tension POAG and primary closed-angle glaucoma were too small to make reliable assessment. With statistical powers to detect the Barbadian effects reaching 100%, the three SNPs had no association with susceptibility to POAG or high-tension POAG in this Korean population.

**Figure 2:**
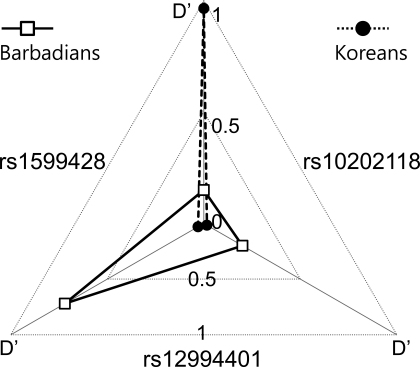
Differences in an LD structure of 2p16.3 between Koreans and Barbadians. All three pairwise LD coefficients (D') among the three documented SNPs, rs12994401, rs1533428, and rs10202118, measured in the Korean control samples of this study (filled circles) are compared with those previously reported for the Barbadian samples [12] (empty squares) in a radial plot.

LD (D’) or correlation (r^2^) coefficients among the three SNPs were substantially different between this Korean population and the Barbadian population (Figure 2). LD between rs12994401 and rs1533428 was high in Barbadians (D’=0.72) [12] but very low in Koreans (D’=0.030, r^2^=0.0010). In contrast, LD between rs1533428 and rs1020118 was low in Barbadians (D’=0.15) [12] but very high in Koreans (D’=0.96, r^2^=0.88). In addition, LD between rs12994401 and rs1020118 was low in Barbadians (D’=0.20) [12] and very low in Koreans (D’=0.016, r^2^=0.000). Furthermore, the correlations among the three SNPs were all low (r^2^≤0.35) in Afro-Americans and Ghanaians [13]. Accordingly, the LD structure around these SNPs appeared much different from population to population.

If a genetic marker correlates highly with a real causative genetic variation in a population but poorly in another population, association tests of the marker would yield inconsistent or contrasting results between the two populations. We therefore aimed to test all the SNPs that correlate considerably with the POAG-associating SNPs in the Barbadian population but not necessarily in the Korean population. Because rs12994401 had a much larger effect than the other two SNPs (OR 35 versus 6.7 and 2.0) in the original study [12], SNPs correlating considerably with rs12994401 rather than with the other two were examined in this study.

We found that rs12994401 correlated with 63 SNPs at r^2^ values ≥0.30 in the African-descendent Yoruba (YRI) population of the International HapMap Project, which could be the closest to Barbadians among the HapMap populations. (None of the three reported SNPs were included in the 63 SNPs, because the correlations among them were poor, 0.001≤r^2^≤0.177 in the YRI population.) These 63 SNPs extended from rs10200429 on chromosome position 46,158,806 (in chromosome 2p21) to rs737366 on position 65,034,194 (in 2p14), spanning a 19-megabase region. This region enclosed all three above-mentioned POAG-linkage loci previously mapped using Caucasian (GLC1H, 2p15–16.2) [10], Chinese (2p14–16.3) [11], and Barbadian (2p15–21) [12] families (Figure 1). Out of 63 SNPs, 42 could be deemed monomorphic or very rare, because no minor allele has been observed for them in the Korean population of the Korean HapMap Project or in the Chinese (CHB) or Japanese (JPT) population of the International HapMap Project.

All participants were then genotyped for the remaining 21 SNPs in this study. Four of them had very rare minor alleles (minor allele frequency ≤0.0013). Among the remaining 17 SNPs (Table 3), none showed significant association with susceptibility to POAG (p≥0.053) in a co-dominant genetic model, although a SNP (rs11681755) showed marginal association (p=0.044) with susceptibility to glaucoma (not POAG). Accordingly, none of the 20 typed polymorphic SNPs listed in Table 2 and Table 3 achieved a significance level of multiple testing (α=0.05/20=0.0025) for association with susceptibility to POAG.

**Table 3:** Association tests for 17 typed SNPs on 2p14–2p21.

** **	**Controls**	**Glaucoma**	**POAG**	**Co-dominant, p†**	
SNP (*A*>*B*)/position*	**MAF/*AA*/*AB*/*BB***	**MAF/*AA*/*AB*/*BB***	**MAF/*AA*/*AB*/*BB***	**Glaucoma**	**POAG**
rs11685904 (*C*>*T*)/48861904	0.060/719/96/1	0.039/188/16/0	0.041/158/14/0	0.289	0.374
rs13024367 (*C*>*G*)/48899516	0.075/717/116/5	0.074/180/27/2	0.077/149/25/1	0.793	0.973
rs17542930 (*T*>*C*)/49086284	0.187/583/245/40	0.198/138/61/11	0.212/112/55/10	0.893	0.599
rs17483987 (*C*>*T*)/49090453	0.158/594/204/29	0.168/143/47/10	0.175/119/41/9	0.661	0.578
rs17831941 (*A*>*G*)/49484456	0.027/641/33/2	0.032/192/13/0	0.029/162/10/0	0.738	0.908
rs13025974 (*T*>*C*)/50135149	0.242/469/286/52	0.274/112/68/21	0.272/94/58/17	0.124	0.224
rs17039430 (*G*>*A*)/50139486	0.244/523/345/52	0.268/121/83/18	0.269/101/67/16	0.304	0.249
rs11681755 (*A*>*G*)/50457643	0.118/635/176/9	0.147/149/45/7	0.153/121/41/5	0.044	0.079
rs727248 (*T*>*C*)/51580556	0.163/614/240/23	0.152/158/52/7	0.142/134/415	0.386	0.360
rs6545379 (*A*>*G*)/54220898	0.074/743/114/7	0.085/173/31/2	0.090/143/27/2	0.715	0.520
rs243016 (*T*>*A*)/60588713	0.141/630/199/20	0.138/158/46/6	0.136/134/36/6	0.882	0.580
rs243052 (*C*>*T*)/60590257	0.143/649/207/22	0.137/159/46/6	0.136/135/36/6	0.880	0.583
rs12476816 (*G*>*A*)/62074097	0.308/412/374/80	0.343/89/111/20	0.341/72/96/14	0.104	0.053
rs7581802 (*A*>*C*)/62089938	0.300/438/397/74	0.330/96/107/20	0.324/79/92/14	0.397	0.351
rs888351 (*T*>*A*)/65026329	0.103/739/157/15	0.109/180/41/4	0.104/151/33/3	0.844	0.991
rs2098480 (*C*>*T*)/65026460	0.097/637/129/11	0.085/141/20/4	0.084/122/18/3	0.333	0.476
rs737366 (*G*>*T*)/65034194	0.102/700/151/13	0.111/166/38/4	0.106/140/31/3	0.804	0.966

*Major allele of each single-nucleotide polymorphism (SNP) is referred to as *A* and minor allele as *B*. Chromosome positions of SNPs are based on the NCBI genome build 37.1. Not listed are rs1020049, rs13396706, rs983511, and rs17049908 with minor allele frequency (MAF) <1%. †The p-values for association with susceptibility to glaucoma or primary open-angle glaucoma (POAG) were calculated according to a co-dominant genetic model using logistic regression with adjustment for age and gender.

To look for association with other SNPs, imputation was performed using the International HapMap Project phase-2 haplotype data for the CHB and JPT populations as templates. Using the genotyping results for all 24 typed SNPs including the four very rare SNPs, the genotypes of 21,201 SNPs located in a 25-megabase region from chromosome 2p14 (chromosome position 68,145,622) to 2p21 (position 43,146,705) were imputed for all participants. Among them, only 4,260 SNPs were imputed to be non-monomorphic (with a minor allele frequency >0) and under Hardy–Weinberg equilibrium (p>0.001).

All 4,260 imputed SNPs were then statistically tested for association with POAG susceptibility (Figure 1). None of them achieved a significance level of multiple testing for association (α=0.05/4,260=1.2 ×10^−5^), although quite many of them could be considered for marginal association. For example, considerably low, but not significant, p values were estimated for four imputed SNPs, rs17039353, rs1875806, rs1317435, and rs6545135 in the order of increasing p values (Table 4), located near the originally reported SNPs in the 2p16.3 locus (Figure 1). Nevertheless, none of the HapMap SNPs located in the extended 2p14–21 chromosomal region appeared to associate with POAG susceptibility.

**Table 4:** Association tests for four imputed SNPs on 2p16.3.

** **	**Controls**	**POAG**	***AB* versus *AA*†**	
SNP (*A*>*B*)/position*	**MAF/*AA*/*AB*/*BB***	**MAF/*AA*/*AB*/*BB***	**OR (95% CI)**	**p**
rs17039353 (*C*>*T*)/50057389	0.0027/928/5/0	0.021/180/8/0	9.28 (2.95–29.17)	0.00013
rs1875806 (*T*>*C*)/49402679	0.0021/929/4/0	0.019/181/7/0	9.29 (2.64–32.49)	0.00051
rs1317435 (*G*>*T*)/52472816	0.033/871/62/0	0.066/163/25/0	2.34 (1.41–3.87)	0.00091
rs6545135 (*C*>*T*)/50052595	0.0016/930/3/0	0.013/183/5/0	10.50 (2.44–45.23)	0.0016

*Major allele of each single-nucleotide polymorphism (SNP) is referred to as *A* and minor allele as *B*. Chromosome positions of SNPs are based on the NCBI genome build 37.1. †Because minor allele frequency (MAF) was low and the minor-allele homozygotes (*BB*) were absent in these four imputed SNPs, odds ratio (OR), 95% confidence interval (CI), and p-values were calculated for the heterozygotes (*AB*) versus the major-allele homozygotes (*AA*) using logistic regression with adjustment for age and gender.

Then, our study samples were stratified according to their ages and genders to examine their effects. The POAG case and control groups were each divided into two subgroups; those who were older than the median age (62.5 years) of the 188 POAG cases and those who were younger. Additionally they were each divided into three subgroups according to the tertile ages of the POAG cases. In any subgroup comparisons, no SNP showed association with POAG susceptibility in logistic regression analyses with adjustment for gender (data not shown). Furthermore, we examined the gender effects by stratifying our POAG case and control samples by gender. However, no SNP showed POAG association in gender-stratified logistic regression with adjustment for age (data not shown). Accordingly, no SNP association with POAG susceptibility was observed in Koreans regardless of age and gender.

## Discussion

We report here that none of the 21,267 SNPs located within a human chromosome region from 2p14 to 2p21 associate with susceptibility to POAG in Koreans. These SNPs were examined in this study, because they could potentially correlate considerably with rs12994401 on 2p16.3, which had very strongly associated with POAG susceptibility in Afro-Caribbean residents of Barbados [12]. This case-control association study using 1,159 unrelated participants had a sufficient statistical power (100%) to detect the Barbadian association. Accordingly, not only the 2p16.3 association was absent in Koreans, but also no SNP markers representing it could be found from an extended chromosomal region 2p14–21.

The contrasting results from the Barbadian and Korean studies were unlikely attributed to the difference in LD structure, which was nevertheless substantial between the two populations as partly described in a preceding section with respect to LD among the three reported SNPs (Figure 2). An explanation could have been that a variation(s) commonly causative in the two populations correlate with rs12994401 in Barbadians considerably enough to show indirect association but not in Koreans. With that possibility in mind, we examined all the HapMap SNPs located within chromosome 2p14–21, a much wider region than 2p16.3, except those that had been demonstrated to correlate poorly with rs12994401 (r^2^<0.3) in an African-descent Yoruba population or to be little polymorphic in a Korean, Chinese or Japanese population.

Although we should not completely rule out a possibility that the 2p16.3 association could be demonstrated by yet another variation, because we did not examine the variations that were not typed in the International HapMap Project and those, if any, that could correlate with rs12994401 poorly in Yorubas but highly in Barbadians, the lack of 2p16.3 association in Ghanaians [13], Japanese [14], and Koreans could arise less likely from LD difference than other possible causes such as sampling biases in the studies.

The age distribution differed substantially between the Korean and Barbadian study subjects. The Barbadian cases were 10.1 years older than the Korean cases on the average (71.0 versus 60.9 years), whereas the Barbadian controls were only slightly older than the Korean controls (61.2 versus 57.6 years). We examined the age effects by stratifying our study samples according to their ages. The POAG case and control groups were each divided into two or three subgroups according to the median or tertile ages of the POAG cases, respectively. In any subgroup comparisons, no SNP showed association with POAG susceptibility in logistic regression analyses with adjustment for gender, suggesting that the age difference rarely caused the contrasting results.

The average IOP levels were comparable between the Barbadian (26.7 mmHg) and Korean (26.4 mmHg) cases, because we primarily recruited high-tension POAG for the cases of this study (77.4% of all cases) unlike the previous Japanese study [14], despite that the prevalence of high-tension POAG (0.8%) is much lower than that of normal-tension POAG (2.7%) in the general population of Koreans [17]. Although gender ratio was not known for the Barbadian samples, we examined the gender effects by stratifying our POAG case and control samples by gender. However, no SNP showed POAG association in gender-stratified logistic regression with adjustment for age. Accordingly, IOP levels and gender may not be the factors attributing to the contrasting results.

If the differences in LD structure, IOP level, age distribution, and gender ratio did not cause the contrasting results between the tested populations, POAG susceptibility localized to the haplotype marked by rs12994401 may be unique to Barbadians and absent in the other tested populations including Koreans, Japanese, and Ghanaians lacking the association, and even in the Afro-American population, where rs12994401 showed only moderate association and the risk-associated allele (C allele) was different from the Barbadian population (T allele).

Alternatively, this haplotype may interact with another genetic, epigenetic or environmental variation that differs between the Barbadian and the other populations, in affecting the susceptibility. Because this chromosomal region has been linked to POAG susceptibility in Caucasian (2p15–16.2) and Chinese (2p14–16.3) families, POAG-associating variations perhaps need to be looked for in Caucasian and Chinese populations.

In this study of Koreans with sufficient power, susceptibility to POAG or high-tension glaucoma did not associate with rs12994401 on 2p16.3 that had shown a very strong association in an Afro-Caribbean population. Many other SNPs within an extended chromosomal region (2p14–21) potentially correlating considerably with rs12994401 were genotyped or imputed, but none of them showed association, quite confidently rejecting the 2p16.3 association with POAG susceptibility in Koreans.
